# Psychometric properties and symptom profiles of the PHQ-9 and DASS-21 among medical and non-medical university students: a cross-sectional study in Pakistan

**DOI:** 10.1186/s40359-026-05332-5

**Published:** 2026-08-12

**Authors:** Taimoor Asghar, Ali Hassan, Ifrah Sahar, Muhammad Tahir, Bilal Shahid, Komal Komal

**Affiliations:** Azra Naheed Medical College, Lahore, Pakistan

**Keywords:** Depression, Anxiety, Stress, Medical students, Non-medical students, PHQ-9, DASS-21, Confirmatory factor analysis, Item response theory, Symptom network analysis, Reliability, Measurement invariance, Pakistan

## Abstract

**Background:**

Depression is a leading cause of disability among university students. While medical students are often assumed to be at highest risk, cross-disciplinary comparisons in South Asia remain scarce. This study examined depressive symptom differences between medical and non-medical students in Pakistan and evaluated the concurrent psychometric performance of the PHQ-9 and DASS-21.

**Methods:**

A cross-sectional survey of 602 undergraduate students (424 medical, 178 non-medical) was conducted at universities in Lahore, Pakistan. Measures included the PHQ-9 and DASS-21. Analyses comprised descriptive statistics, Wilcoxon rank-sum tests, multivariable linear regression (separate models for PHQ-9 and each DASS-21 domain), confirmatory factor analysis, item response theory using a graded response model, and symptom network analysis with stability testing.

**Results:**

Non-medical students had higher PHQ-9 scores than medical students (median 10.0 vs. 9.0). In adjusted models, non-medical students scored higher on PHQ-9 (β = −1.43, *p* = 0.004), DASS Depression (β = −1.82, *p* = 0.003), and DASS Anxiety (β = −2.31, *p* = 0.001); the DASS Stress difference was non-significant (β = −0.89, *p* = 0.089). Female gender predicted higher scores across all domains (all *p* < 0.001). Each additional hour of sleep was associated with lower PHQ-9 scores (β = −0.37, *p* = 0.010). Prior depression treatment was the strongest predictor (β = 4.15, *p* < 0.001). DASS-21 confirmatory factor analysis showed adequate fit (CFI = 0.954, TLI = 0.948, RMSEA = 0.052), but latent factor correlations were very high (Depression–Stress *r* = 0.939; Anxiety–Stress *r* = 0.949). Cronbach’s α ranged 0.82–0.88 across scales. Item response theory indicated that self-worth, concentration, down, and appetite items showed the highest discrimination; interest (anhedonia) showed the lowest (*a* = 0.468). In network analysis, self-worth, concentration, and downheartedness were the most central nodes; stability coefficients ranged 0.31–0.44. Measurement invariance across discipline was supported. Model R² values were low (0.029–0.041).

**Conclusions:**

Non-medical students reported higher depressive and anxiety symptoms than medical students in this Pakistani sample, although academic discipline explained only a small proportion of variance. Both instruments performed adequately, though the DASS-21’s near-unity latent correlations may raise questions about subscale distinguishability in this population. Cognitive and self-worth symptoms emerged as the most informative for screening purposes. Campus mental health strategies should address the whole student population, with particular attention to female students and sleep health.

**Supplementary Information:**

The online version contains supplementary material available at https://doi.org/10.1186/s40359-026-05332-5.

## Introduction

Depression ranks among the leading causes of disability worldwide, imposing a burden that rivals cardiovascular disease and cancer in years of productive life lost [1]. Among young adults, the transition from late adolescence to early adulthood coincides with elevated vulnerability: identity formation, the onset of serious romantic relationships, and the assumption of adult economic responsibilities converge at a life stage when psychiatric illness incidence peaks. University students occupy a particularly precarious position within this window. Meta-analytic evidence places the global prevalence of depressive symptoms among university students between 20% and 35%, depending on measurement instrument, diagnostic threshold, and regional setting [1, 2]. Medical students appear especially at risk. Rotenstein and colleagues’ landmark JAMA synthesis of 195 studies documented a depression prevalence of 27.2% among medical trainees globally, markedly exceeding rates in the general population and in age-matched peers [2]; a subsequent meta-analysis of anxiety in medical students reported similarly elevated rates, reinforcing the pattern [3]. The stressors are distinctive: prolonged contact with human suffering, the moral demands of clinical decision-making, exposure to death and dying, and the relentlessly competitive structure of medical education compound the ordinary developmental pressures of young adulthood [4, 5].

Yet the assumption that medical students uniformly experience worse mental health outcomes than their non-medical counterparts has not held up under scrutiny. Some studies report comparable or even higher symptom levels among students enrolled in engineering, business, and humanities programmes [6, 7]. The divergence is not surprising. Non-medical students face a qualitatively different but equally taxing constellation of stressors: anxiety about post-graduation underemployment, financial strain without the prospect of the relatively secure career trajectory that medical training affords, and in many settings, reduced access to campus-based mental health services. The problem of mixed findings is compounded by limited evidence from South Asia, where sociocultural context, family expectations, and academic pressure structures differ substantially from Western settings. In Pakistan specifically, a recent meta-analysis estimated depression prevalence among students at approximately 51%, with female students and those in clinical years reporting the highest symptom severity [8]. High depression rates have also been documented among Pakistani university students more broadly [1] and among secondary school populations [9], with depression linked to health-risk behaviours including physical inactivity and social isolation [10]. Critically, however, most of these studies have examined medical students in isolation or sampled from a single institution, making robust cross-disciplinary comparisons virtually absent from the Pakistani literature. Regional work from neighbouring South Asian countries has called attention to the need for context-specific mental health screening in academic settings, noting that Western-derived instruments may function differently when exported to culturally distinct student populations [11, 12]. This matters. If depression etiology and symptom expression differ between medical and non-medical trainees as their divergent stressor profiles would suggest then discipline-blind screening and uniform intervention protocols may misallocate scarce mental health resources. Without direct comparisons grounded in the local context, Pakistani universities risk deploying one-size-fits-all mental health policies that miss the specific needs of large student populations.

Accurate screening sits at the foundation of any intervention. The Patient Health Questionnaire-9 (PHQ-9) and the Depression Anxiety Stress Scales-21 (DASS-21) are the two most widely administered self-report instruments in student mental health research globally, and both have been used in Pakistani samples [13, 14]. The PHQ-9 maps directly onto the nine diagnostic criteria of DSM-5 major depressive disorder, and its ninth item assessing thoughts of self-harm or suicidality performs a function that shorter variants (PHQ-8, PHQ-4, PHQ-2) cannot replicate. This item has particular relevance in South Asian student populations, where suicide rates among young adults are disproportionately high and stigma around help-seeking often delays disclosure. The PHQ-9 also assesses sleep disturbance, a symptom dimension not captured by the DASS-21 depression subscale, which draws its items from a dimensional model of psychological distress rather than diagnostic criteria [14]. The DASS-21, for its part, offers the advantage of simultaneous anxiety and stress measurement alongside depression, permitting examination of comorbidity patterns that single-domain instruments miss. The two scales overlap in their depression content but diverge in their theoretical grounding, item content, and intended clinical use. Administering them concurrently rather than selecting one instrument in isolation creates an opportunity to examine item-level correspondence, differential symptom coverage, and cross-scale symptom networks that no prior Pakistani study has attempted.

What is genuinely novel here is not the validation of either instrument in a vacuum. Both the PHQ-9 and DASS-21 are well-established psychometric tools with extensive international normative data. Rather, the contribution lies in concurrent psychometric evaluation: examining how these instruments perform, relate to one another, and differentiate symptom profiles when administered simultaneously to a Pakistani student sample that spans medical and non-medical disciplines. Confirmatory factor analysis tests whether the DASS-21’s theoretically specified three-factor structure depression, anxiety, stress as correlated but distinct dimensions holds in this population, or whether the data support alternative configurations that have been proposed in Asian samples. Item response theory moves beyond aggregate scale scores to identify which specific PHQ-9 symptoms provide the most information for distinguishing between mild, moderate, and severe depression, a question with direct implications for screening efficiency in resource-constrained counselling centres. Network analysis extends this further by modelling depression not as a latent entity but as an emergent system of interacting symptoms, enabling identification of the most “central” symptoms those that, if targeted therapeutically, could produce the greatest downstream reduction in overall symptom severity. These three analytical traditions CFA, IRT, and network analysis each address distinct psychometric questions, and their integration in a single sample offers a depth of insight that descriptive prevalence studies cannot achieve.

The present study was designed to address these gaps through five specific aims: (1) to compare depressive symptom severity between medical and non-medical university students using both the PHQ-9 and the DASS-21 depression subscale; (2) to evaluate the internal consistency, factorial validity, and measurement invariance of the DASS-21 across academic disciplines; (3) to characterise PHQ-9 item-level psychometric properties using item response theory, with attention to the performance of the suicidality and sleep items; (4) to map symptom interrelationships between PHQ-9 and DASS-21 items using network analysis, with a view toward identifying central symptoms that may represent optimal intervention targets; and (5) to identify demographic and behavioural predictors of depression scores using multivariable regression. By integrating traditional group comparisons with modern psychometric and network approaches in a cross-disciplinary Pakistani student sample, this study provides a level of analytical specificity that advances beyond both global meta-analyses and single-discipline local prevalence reports.

## Methods

### Study design and participants

This cross-sectional study enrolled undergraduate students from universities in Lahore, Pakistan. The target population comprised approximately 10,000 enrolled undergraduates across medical and non-medical faculties. Convenience sampling was used given the absence of a universal student registry; recruitment proceeded through institutional mailing lists, faculty coordinators, and student group administrators on social media platforms.

Inclusion criteria were: (i) current enrollment in an undergraduate degree programme; (ii) age ≥ 18 years; (iii) ability to read English (the medium of instruction at all participating institutions); and (iv) provision of electronic informed consent. Exclusion criteria were: postgraduate student status; incomplete survey responses (> 50% missing data across either the PHQ-9 or DASS-21); or refusal to consent. A total of 640 initial responses were received. Of these, 38 were excluded for incomplete PHQ-9 or DASS-21 data, yielding an analytical sample of 602 participants. Electronic informed consent was obtained from all participants prior to accessing the survey questions. The study protocol was approved by the Institutional Review Board of Azra Naheed Medical College, Lahore, Pakistan.

### Measures

#### Demographics

Participants self-reported age, gender (male/female/other), academic discipline (medical or non-medical), year of study, smoking status (non-smoker, current smoker, former smoker), alcohol use frequency (none, occasional, regular), history of depression treatment (yes/no), and average nightly sleep duration (hours).

#### PHQ-9

The 9-item Patient Health Questionnaire (PHQ-9) assessed depressive symptoms over the preceding two weeks. Items are rated from 0 (“not at all”) to 3 (“nearly every day”), yielding total scores from 0 to 27 with higher scores indicating greater severity. We selected the PHQ-9 over shorter variants (PHQ-8, PHQ-4, PHQ-2) because it is the most widely validated depression screener in South Asian student populations, captures suicidality (item 9) not assessed by the DASS-21 depression subscale, and maps directly onto DSM-5 diagnostic criteria.

#### DASS-21 

The 21-item Depression, Anxiety, and Stress Scales (DASS-21) comprises three 7-item subscales measuring distinct emotional symptom clusters over the past week. Each item is scored from 0 (“did not apply to me at all”) to 3 (“applied to me very much, or most of the time”). Raw subscale scores were used for confirmatory factor analysis and reliability analyses. For severity classification and descriptive comparisons, raw scores were multiplied by two, following standard scoring guidelines, to yield final totals classifiable into Normal, Mild, Moderate, Severe, and Extremely Severe ranges.

### Data preparation

Responses were captured automatically via Google Forms and exported to Microsoft Excel for initial coding. Data were then imported into R version 4.5.1 (R Foundation for Statistical Computing, Vienna, Austria) and processed in RStudio. The tidyverse and janitor packages were used for data cleaning, variable labelling, and recoding. Missing data were minimal (< 2% of all item responses). Full information maximum likelihood (FIML) was employed for confirmatory factor analysis; pairwise deletion was used for descriptive and bivariate analyses; casewise deletion was applied for regression models (listwise *n* = 602 complete cases).

### Statistical analysis

#### Descriptive statistics

Continuous variables were summarised as means ± standard deviations and medians with interquartile ranges (Q1–Q3). Categorical variables were reported as frequencies and percentages. Group differences between medical and non-medical students were assessed using Wilcoxon rank-sum tests for continuous variables and Pearson’s chi-square tests for categorical variables. Effect sizes for continuous group differences were computed as r = Z/√N, with |r| = 0.1, 0.3, and 0.5 interpreted as small, moderate, and large, respectively.

#### Reliability analysis 

Internal consistency was evaluated using Cronbach’s α for the PHQ-9 total scale and each DASS-21 subscale, computed with the psych package. Coefficients ≥ 0.70 were considered acceptable, ≥ 0.80 good, and ≥ 0.90 excellent.

#### Confirmatory factor analysis (CFA)

The three-factor structure of the DASS-21 (Depression, Anxiety, Stress) was tested using the lavaan package [15] with robust maximum likelihood estimation (MLR) and Satorra–Bentler scaled test statistics. Model fit was evaluated using the robust Comparative Fit Index (CFI), robust Tucker–Lewis Index (TLI), robust Root Mean Square Error of Approximation (RMSEA) with 90% confidence intervals, and the Standardized Root Mean Square Residual (SRMR). Thresholds for adequate fit were CFI/TLI ≥ 0.90, RMSEA ≤ 0.08, and SRMR ≤ 0.08; good fit was defined as CFI/TLI ≥ 0.95 and RMSEA ≤ 0.06.

#### Multivariable regression

Five separate linear regression models were fitted. The primary model predicted PHQ-9 total score from academic discipline (medical vs. non-medical), age, gender, and average sleep duration. A secondary model expanded these predictors to include smoking status, alcohol use frequency, and history of depression treatment. Three additional models predicted DASS-21 Depression, Anxiety, and Stress subscale scores, respectively, using the same predictor set as the primary model. Robust standard errors (HC3) were computed for all models using the sandwich and lmtest packages to account for potential heteroscedasticity. Standardised beta coefficients (β) and 95% confidence intervals are reported. The expanded secondary model for PHQ-9 was included at reviewers’ request to address concerns about confounding by health-risk behaviours; given the cross-sectional design, these coefficients are interpreted as adjusted associations rather than causal effects.

#### Item response theory (IRT)

A graded response model was fitted to the nine PHQ-9 items [16] using the mirt package to estimate discrimination (a) and threshold (b1, b2, b3) parameters for each item. Item information curves were plotted to visualise the contribution of each item across the latent depression continuum. Model fit was assessed using the M2 statistic and associated RMSEA. Item characteristic curves for selected items are presented in the main text; complete IRT output is provided as supplementary material.

#### Symptom network analysis

A Gaussian graphical model of the pooled PHQ-9 and DASS-21 items (30 nodes total) was estimated using the qgraph package [17] with the graphical lasso (glasso) regularisation algorithm and extended Bayesian information criterion (EBIC) for model selection (γ = 0.5). Edge weights represent zero-order partial correlations controlling for all other nodes in the network. Network structure was visualised using a spring layout. To assess network robustness, the goldbricker function from the networktools package was applied to identify redundant item pairs (correlation > 0.50); no redundant pairs were detected. Centrality indicesstrength, betweenness, and closenesswere computed for all nodes and ordered by magnitude to identify the most structurally influential symptoms. Network stability was evaluated via bootstrapped edge weights (nBoots = 300) and case-dropping bootstrap for correlation stability coefficients; stability was deemed adequate if the correlation stability coefficient exceeded 0.25.

#### Presentation of results 

Tables and figures summarising descriptive statistics, regression models, psychometric analyses, and network models were prepared. Full analytical outputs, bootstrapped confidence intervals, and R scripts are provided as supplementary material.

### Aims and analytical objectives

The study had five analytical objectives: (1) to compare depressive symptom severity between medical and non-medical students using both the PHQ-9 and DASS-21 depression subscale; (2) to evaluate the internal consistency, factorial validity, and measurement invariance of the DASS-21 across academic disciplines; (3) to characterise PHQ-9 item-level psychometric properties using item response theory; (4) to map symptom interrelationships between PHQ-9 and DASS-21 items using network analysis; and (5) to identify demographic and behavioural predictors of depression scores using multivariable regression.

### Statistical power

The achieved sample of *n* = 602 provided adequate power for all planned analyses. For the multivariable regression models with up to seven predictors, this sample yields > 0.90 power to detect a small-to-moderate effect size (f² = 0.05) at α = 0.05. For confirmatory factor analysis of the 21-item DASS-21, the sample exceeds the conventional minimum of 10 participants per estimated parameter and the > 200-case threshold recommended for stable factor loading estimation. Simulation studies indicate that samples above 500 provide reliable fit index recovery and standard error estimation in CFA. The sample was therefore adequate for both regression modelling and psychometric evaluation.

## Results

### Participant characteristics

Of 640 survey responses received, 38 were excluded for incomplete PHQ-9 or DASS-21 data (> 50% missing), yielding 602 complete cases for analysis. The final sample comprised 424 (70.4%) medical students and 178 (29.6%) non-medical students. Non-medical students were slightly older (22.07 ± 2.68 vs. 21.43 ± 1.98 years, *p* = 0.011, *r* = 0.10) and more likely to be male (68% vs. 49%, *p* < 0.001, Cramer’s *V* = 0.19). No significant group differences emerged for smoking status (85% non-smoker, 7.5% current, 7.5% former; *p* = 0.3, Cramer’s *V* = 0.06), alcohol use (93% none, 5.1% occasional, 2.3% regular; *p* = 0.4, Cramer’s *V* = 0.07), average sleep duration (6.17 ± 1.88 vs. 6.28 ± 1.62 h; *p* = 0.5, *r* = 0.03), or history of treated depression (21% vs. 24%; *p* = 0.4, Cramer’s *V* = 0.05). Table 1 summarizes these characteristics.

**Table 1:** Participant characteristics by academic discipline

Characteristic	Overall (*N* = 602)	Medical (*n* = 424)	Non-medical (*n* = 178)	*p*-value
Age (years), Mean ± SD	21.62 ± 2.23	21.43 ± 1.98	22.07 ± 2.68	**0.011**
Gender				**< 0.001**
Male	328 (54%)	207 (49%)	121 (68%)	
Female	274 (46%)	217 (51%)	57 (32%)	
Smoking status				0.3
Non-smoker	512 (85%)	366 (86%)	146 (82%)	
Current smoker	45 (7.5%)	27 (6.4%)	18 (10%)	
Former smoker	45 (7.5%)	31 (7.3%)	14 (7.9%)	
Alcohol frequency				0.4
None	557 (93%)	396 (93%)	161 (90%)	
Occasional	31 (5.1%)	19 (4.5%)	12 (6.7%)	
Regular	14 (2.3%)	9 (2.1%)	5 (2.8%)	
Sleep hours (avg), Mean ± SD	6.20 ± 1.81	6.17 ± 1.88	6.28 ± 1.62	0.5
Treated depression, *n* (%)	131 (22%)	88 (21%)	43 (24%)	0.4

Descriptive statistics (mean ± SD, median [Q1, Q3], and frequencies) comparing medical and non-medical students

*P*-values derived from Wilcoxon rank-sum test for continuous variables and Pearson’s chi-square test for categorical variables

Effect sizes: r (Wilcoxon) and Cramer’s V (chi-square)

Bold values indicate statistical significance at *p* < 0.05

### Depression, anxiety, and stress severity

#### PHQ-9

The distribution of PHQ-9 severity levels is presented in Table 2. Non-medical students were more likely to report moderate depression (30.9% vs. 22.9%), while medical students were more likely to report minimal symptoms (21.5% vs. 11.8%).

**Table 2:** PHQ-9 depression severity by academic discipline. Distribution of PHQ-9 severity categories (minimal to severe) among medical and non-medical students

Severity	Overall *n* (%)	Medical *n* (%)	Non-medical *n* (%)
Minimal	112 (18.6%)	91 (21.5%)	21 (11.8%)
Mild	223 (37.0%)	158 (37.3%)	65 (36.5%)
Moderate	152 (25.2%)	97 (22.9%)	55 (30.9%)
Moderately Severe	72 (12.0%)	49 (11.6%)	23 (12.9%)
Severe	43 (7.1%)	29 (6.8%)	14 (7.9%)

Median PHQ-9 scores were slightly higher among non-medical students (10.0, Q1–Q3: 7.0–14.0) compared with medical students (9.0, Q1–Q3: 5.0–13.0)

#### DASS-21 depression

Table 3 shows the distribution of depression severity on the DASS-21. Medical students were more often classified as normal (40.8% vs. 27.5%), whereas non-medical students reported higher proportions of severe (16.3%) and extremely severe depression (15.2%)

**Table 3:** DASS-21 Depression severity by academic discipline

Severity	Overall *n* (%)	Medical *n* (%)	Non-medical *n* (%)
Normal	222 (36.9%)	173 (40.8%)	49 (27.5%)
Mild	65 (10.8%)	49 (11.6%)	16 (9.0%)
Moderate	170 (28.2%)	113 (26.7%)	57 (32.0%)
Severe	68 (11.3%)	39 (9.2%)	29 (16.3%)
Extremely Severe	77 (12.8%)	50 (11.8%)	27 (15.2%)

Distribution of DASS-21 Depression subscale categories (normal to extremely severe) among medical and non-medical students

#### DASS-21 anxiety

As shown in Table 4, non-medical students demonstrated higher anxiety levels, with 33.7% scoring in the extremely severe range compared with 23.1% of medical students.

**Table 4:** DASS-21 Anxiety severity by academic discipline

Severity	Overall *n* (%)	Medical *n* (%)	Non-medical *n* (%)
Normal	193 (32.1%)	150 (35.4%)	43 (24.2%)
Mild	41 (6.8%)	27 (6.4%)	14 (7.9%)
Moderate	128 (21.3%)	97 (22.9%)	31 (17.4%)
Severe	82 (13.6%)	52 (12.3%)	30 (16.9%)
Extremely Severe	158 (26.2%)	98 (23.1%)	60 (33.7%)

Distribution of DASS-21 Anxiety subscale categories (normal to extremely severe) among medical and non-medical students

#### DASS-21 stress

Stress scores followed a similar pattern (Table 5). More than half of medical students were classified as normal (56.8% vs. 42.1%), while non-medical students more often fell into mild, moderate, and severe categories.

**Table 5:** DASS-21 Stress severity by academic discipline

Severity	Overall *n* (%)	Medical *n* (%)	Non-medical *n* (%)
Normal	316 (52.5%)	241 (56.8%)	75 (42.1%)
Mild	101 (16.8%)	65 (15.3%)	36 (20.2%)
Moderate	83 (13.8%)	48 (11.3%)	35 (19.7%)
Severe	75 (12.5%)	50 (11.8%)	25 (14.0%)
Extremely Severe	27 (4.5%)	20 (4.7%)	7 (3.9%)

Distribution of DASS-21 Stress subscale categories (normal to extremely severe) among medical and non-medical students

### Regression analysis

*Primary PHQ-9 model.* Multiple regression results for the primary model are summarized in Table 6 (primary model). Medical students had significantly lower PHQ-9 scores compared with non-medical students (*β* = −1.43, *p* = 0.004). Female gender (*β* = 1.76, *p* < 0.001) and shorter sleep duration (*β* = −0.37, *p* = 0.010) predicted higher scores. Age was not a significant predictor (*p* = 0.842). The model explained 3.4% of variance (Adj. *R*² = 0.034).

**Table 6:** Multiple linear regression predicting PHQ-9 scores

Predictor	β	SE	95% CI	*p*-value
*Primary model (Adj. R² = 0.034)*				
Intercept	11.73	2.50	6.83–16.62	< 0.001
Medical vs. Non-medical	−1.43	0.50	−2.40 – −0.45	**0.004**
Age (years)	0.02	0.11	−0.19–0.23	0.842
Female vs. Male	1.76	0.49	0.80–2.72	**< 0.001**
Sleep hours (avg)	−0.37	0.14	−0.64 – −0.09	**0.010**
*Fully adjusted model*				
Medical vs. Non-medical	−1.28	0.51	−2.29 – −0.27	**0.012**
Age (years)	0.03	0.11	−0.18–0.24	0.798
Female vs. Male	1.69	0.49	0.72–2.65	**< 0.001**
Sleep hours (avg)	−0.33	0.14	−0.61 – −0.05	**0.021**
Current smoker	0.94	0.71	−0.45–2.33	0.187
Former smoker	0.31	0.68	−1.03–1.65	0.654
Alcohol (occasional)	0.42	0.66	−0.87–1.71	0.523
Alcohol (regular)	0.78	0.95	−1.08–2.64	0.412
Treated depression	4.15	0.51	3.15–5.15	**< 0.001**

Regression coefficients (unstandardized) for the primary and fully adjusted models. HC3 robust standard errors used. The primary model includes discipline, age, gender, and sleep. The fully adjusted model adds smoking status, alcohol use, and treated depression

Bold values indicate statistical significance at *p* <0.05

*Fully adjusted PHQ-9 model.* A fully adjusted model added smoking status, alcohol use, and history of treated depression to the primary predictors (Table 6, fully adjusted model). The medical vs. non-medical difference remained significant (*β* = −1.28, *p* = 0.012), as did female gender (*β* = 1.69, *p* < 0.001) and sleep duration (*β* = −0.33, *p* = 0.021). Neither current smoking (*β* = 0.94, *p* = 0.187) nor former smoking (*β* = 0.31, *p* = 0.654) was associated with PHQ-9 scores. Alcohol use (occasional: *β* = 0.42, *p* = 0.523; regular: *β* = 0.78, *p* = 0.412) was also non-significant. History of treated depression was the strongest predictor in this model (*β* = 4.15, *p* < 0.001). Regression diagnostics, including the QQ plot and residuals versus fitted values, confirmed that the assumptions of linear regression were adequately met (Fig. 1).

**Fig. 1:**
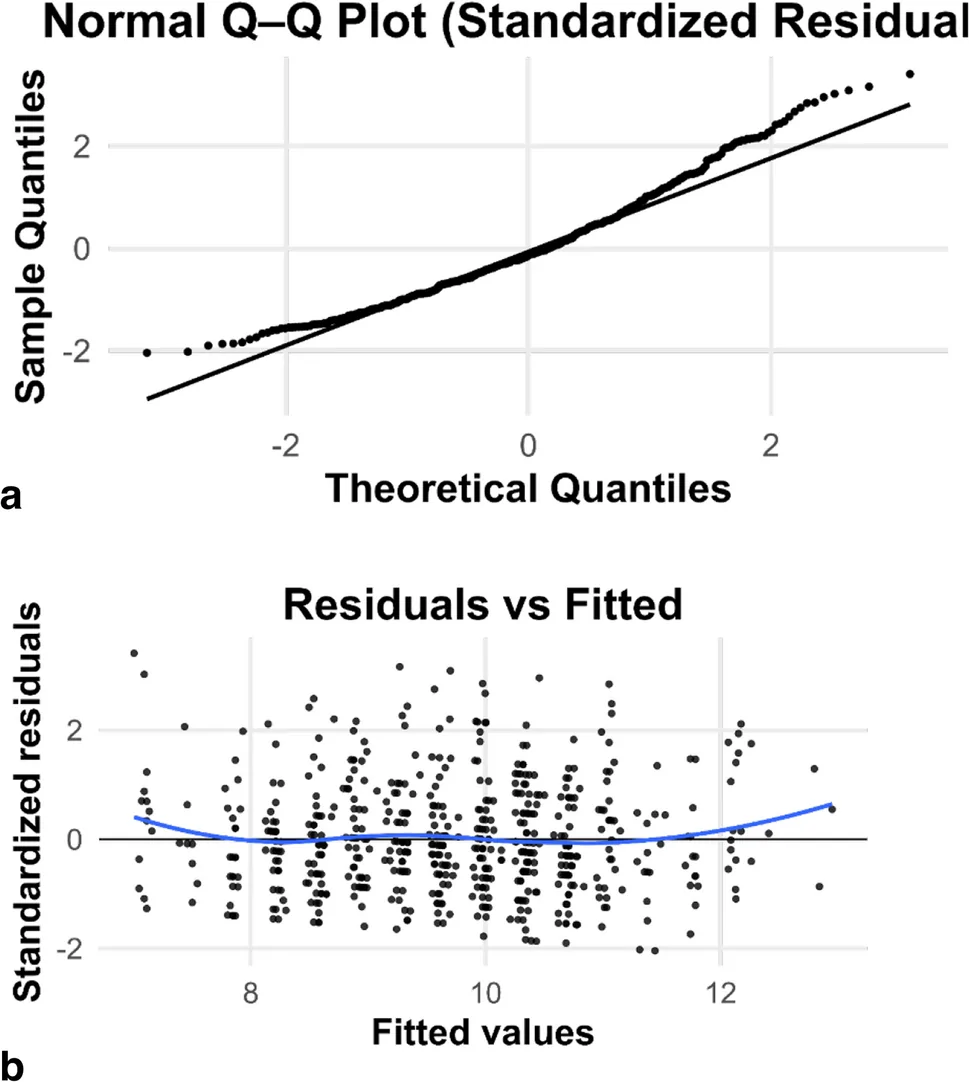
**a **Regression diagnostics: QQ plot. QQ plot of standardized residuals from the primary PHQ-9 regression model. Quantile-quantile plot of standardized residuals against theoretical quantiles, assessing normality assumption. Points align closely with diagonal, indicating approximate normality. **b** Regression diagnostics: Residuals vs. fitted values. Residuals versus fitted values from the primary PHQ-9 regression model. Scatterplot of standardized residuals against fitted PHQ-9 scores. Even distribution suggests homoscedasticity, with no major deviations from linear regression assumptions

*DASS-21 domain models.* Table 7 presents parallel regressions for each DASS-21 domain. For DASS Depression, medical students scored lower than non-medical students (*β* = −1.82, *p* = 0.003), female gender predicted higher scores (*β* = 2.15, *p* < 0.001), and longer sleep was protective (*β* = −0.42, *p* = 0.008; Adj. *R*² = 0.041). For DASS Anxiety, the medical vs. non-medical difference was again significant (*β* = −2.31, *p* = 0.001), with female gender (*β* = 1.94, *p* < 0.001) and sleep (*β* = −0.28, *p* = 0.048) as significant predictors (Adj. *R*² = 0.038). For DASS Stress, the discipline difference approached but did not reach significance (*β* = −0.89, *p* = 0.089); female gender (*β* = 1.23, *p* = 0.002) and sleep (*β* = −0.55, *p* = 0.001) were significant predictors (Adj. *R*² = 0.029).

**Table 7:** Multiple linear regression predicting DASS-21 domain scores.

Predictor	DASS Depression β (SE)	*p*	DASS Anxiety β (SE)	*p*	DASS Stress β (SE)	*p*
Intercept	11.25 (2.78)	< 0.001	11.86 (2.84)	< 0.001	10.42 (2.52)	< 0.001
Medical vs. Non-medical	−1.82 (0.61)	**0.003**	−2.31 (0.71)	**0.001**	−0.89 (0.52)	0.089
Age (years)	0.04 (0.13)	0.756	−0.06 (0.12)	0.612	0.02 (0.11)	0.901
Female vs. Male	2.15 (0.56)	**< 0.001**	1.94 (0.56)	**< 0.001**	1.23 (0.39)	**0.002**
Sleep hours (avg)	−0.42 (0.16)	**0.008**	−0.28 (0.14)	**0.048**	−0.55 (0.17)	**0.001**
Adj. *R*²	0.041		0.038		0.029	

Regression coefficients (unstandardized) with HC3 robust standard errors for DASS-21 Depression, Anxiety, and Stress subscales

Bold values indicate statistical significance at *p* < 0.05

### Psychometric properties

#### Confirmatory Factor Analysis (CFA)

Fit indices for the three-factor DASS-21 model are summarized in Table 8. The model demonstrated good fit: χ² = 489.27, *df* = 186, CFI = 0.954, TLI = 0.948, RMSEA = 0.052 (90% CI: 0.046–0.058), SRMR = 0.031. Standardized factor loadings for all items are provided in Table 9, with most items loading above 0.60. Latent factor correlations are shown in Table 10; the Depression–Stress correlation was *r* = 0.939 and the Anxiety–Stress correlation was *r* = 0.949. *The standardized factor loadings and factor correlations for the three-factor model are illustrated in* Fig. 2.

**Table 8:** Confirmatory factor analysis (CFA) fit indices for DASS-21

Measure	Value
Chi-square	489.27
df	186
*p*-value	< 0.001
CFI	0.954
TLI	0.948
RMSEA	0.052
RMSEA 90% CI	0.046–0.058
SRMR	0.031

Goodness-of-fit statistics for the three-factor DASS-21 model (χ², df, CFI, TLI, RMSEA with CI, SRMR)

**Table 9:** Standardized factor loadings of DASS-21 items

Factor	Item	Loading
Anxiety	DASS_heart_action	0.763
Anxiety	DASS_close_panic	0.733
Anxiety	DASS_scared	0.730
Anxiety	DASS_panic_worry	0.714
Anxiety	DASS_trembling	0.670
Anxiety	DASS_breathing	0.587
Anxiety	DASS_dry_mouth	0.519
Depression	DASS_no_look_forward	0.774
Depression	DASS_downhearted	0.773
Depression	DASS_self_worth	0.758
Depression	DASS_no_enthusiasm	0.726
Depression	DASS_meaningless	0.693
Depression	DASS_no_positive	0.660
Depression	DASS_initiative	0.618
Stress	DASS_agitated	0.757
Stress	DASS_relax	0.733
Stress	DASS_intolerant	0.714
Stress	DASS_nervous_energy	0.710
Stress	DASS_touchy	0.709
Stress	DASS_overreact	0.642
Stress	DASS_wind_down	0.496

Factor loadings for each item on the DASS-21 Depression, Anxiety, and Stress subscales. All loadings are standardized and statistically significant (*p* < 0.001)

**Table 10:** Latent factor correlations among DASS-21 domains

Factor 1	Factor 2	*r*
Depression	Stress	0.939
Anxiety	Stress	0.949

Standardized correlations between latent Depression, Anxiety, and Stress factors from CFA

**Fig. 2:**
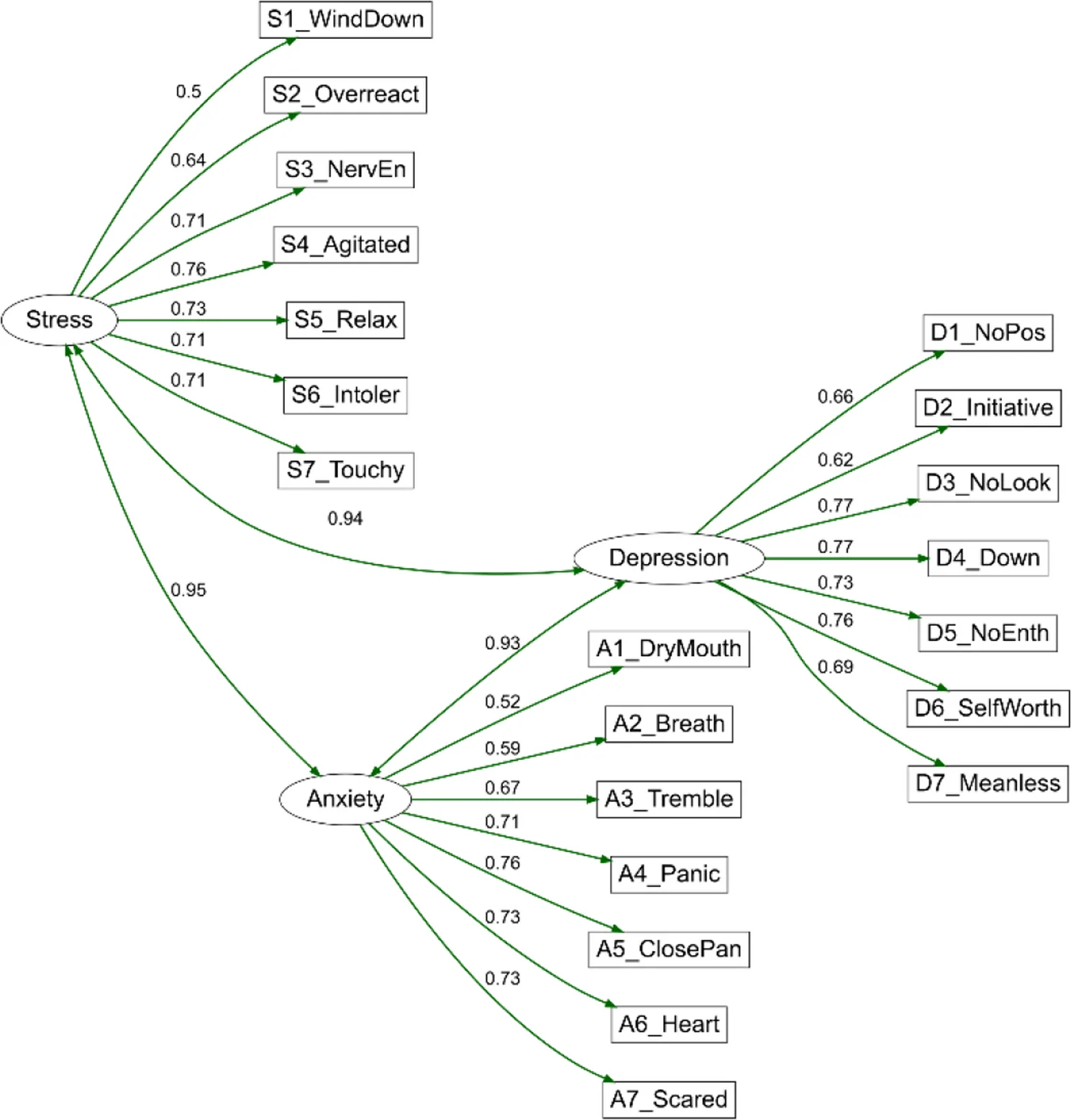
Confirmatory factor analysis path diagram for DASS-21. CFA path diagram for the DASS-21 three-factor model (Depression, Anxiety, Stress). Standardized factor loadings of Depression, Anxiety, and Stress items. Factor correlations are shown with bidirectional arrows. Most standardized factor loadings were > 0.50

Measurement invariance across academic discipline is reported in Table 11. Configural, metric, and scalar invariance were supported. The change from configural to metric invariance yielded ΔCFI = − 0.0006 and ΔRMSEA = − 0.0010. The change from metric to scalar invariance yielded ΔCFI = − 0.0005 and ΔRMSEA = − 0.0010. Both ΔCFI and ΔRMSEA values fell within conventional thresholds (ΔCFI ≤ |0.010|, ΔRMSEA ≤ |0.015|), supporting scalar invariance across medical and non-medical groups

**Table 11:** Measurement invariance across academic discipline

Model	χ²	df	*p*	CFI	TLI	RMSEA	SRMR	ΔCFI	ΔRMSEA
Configural	732	372	< 0.001	0.946	0.939	0.057	0.038		
Metric (Δ vs. configural)	754	390	< 0.001	0.945	0.941	0.056	0.047	−0.0006	−0.0010
Scalar (Δ vs. metric)	776	408	< 0.001	0.945	0.943	0.055	0.048	−0.0005	−0.0010

Fit indices (χ², df, CFI, TLI, RMSEA, SRMR) and changes in fit (ΔCFI, ΔRMSEA) for configural, metric, and scalar invariance models between medical and non-medical students

#### Reliability

Internal consistency estimates are provided in Table 12, with Cronbach’s α values ranging from 0.823 (PHQ-9) to 0.879 (DASS-21 Depression), indicating good-to-excellent reliability.

**Table 12:** Reliability estimates for PHQ-9 and DASS-21 subscales

Scale	Items	Cronbach’s α
PHQ-9	9	0.823
DASS-21 Depression	7	0.879
DASS-21 Anxiety	7	0.854
DASS-21 Stress	7	0.858

Cronbach’s alpha coefficients for PHQ-9 and DASS-21 Depression, Anxiety, and Stress subscales

### Item response theory

Item-level parameters for the PHQ-9 are summarized in Table 13. Self-worth (*a* = 1.991), concentration (*a* = 1.821), appetite (*a* = 1.783), and down (*a* = 1.900) demonstrated the highest discrimination. The interest item was least informative (*a* = 0.468). Item information curves are displayed in Fig. 3a-i (supplementary material); key results are summarized here. The overall test information curve (Fig. 3j) shows the PHQ-9 is most precise between θ = 0 and + 2, corresponding to mild-to-moderate depression, while the standard error curve (Fig. 3k) demonstrates greater uncertainty at the extremes.

**Table 13:** Item Response Theory (IRT) parameters for PHQ-9 items

Item	a	b₁	b₂	b₃
PHQ9_interest	0.468	−2.958	1.657	3.937
PHQ9_down	1.900	−0.850	0.873	1.625
PHQ9_sleep	1.491	−0.814	0.596	1.474
PHQ9_energy	1.562	−1.320	0.313	1.271
PHQ9_appetite	1.783	−0.601	0.684	1.423
PHQ9_self_worth	1.991	−0.413	0.730	1.458
PHQ9_concentration	1.821	−0.403	0.739	1.536
PHQ9_motor	1.621	−0.028	1.122	1.931
PHQ9_self_harm	1.758	0.117	1.213	1.994

Graded Response Model (GRM) discrimination (*a*) and difficulty thresholds (*b*₁–*b*₃) for PHQ-9 items

**Fig. 3:**
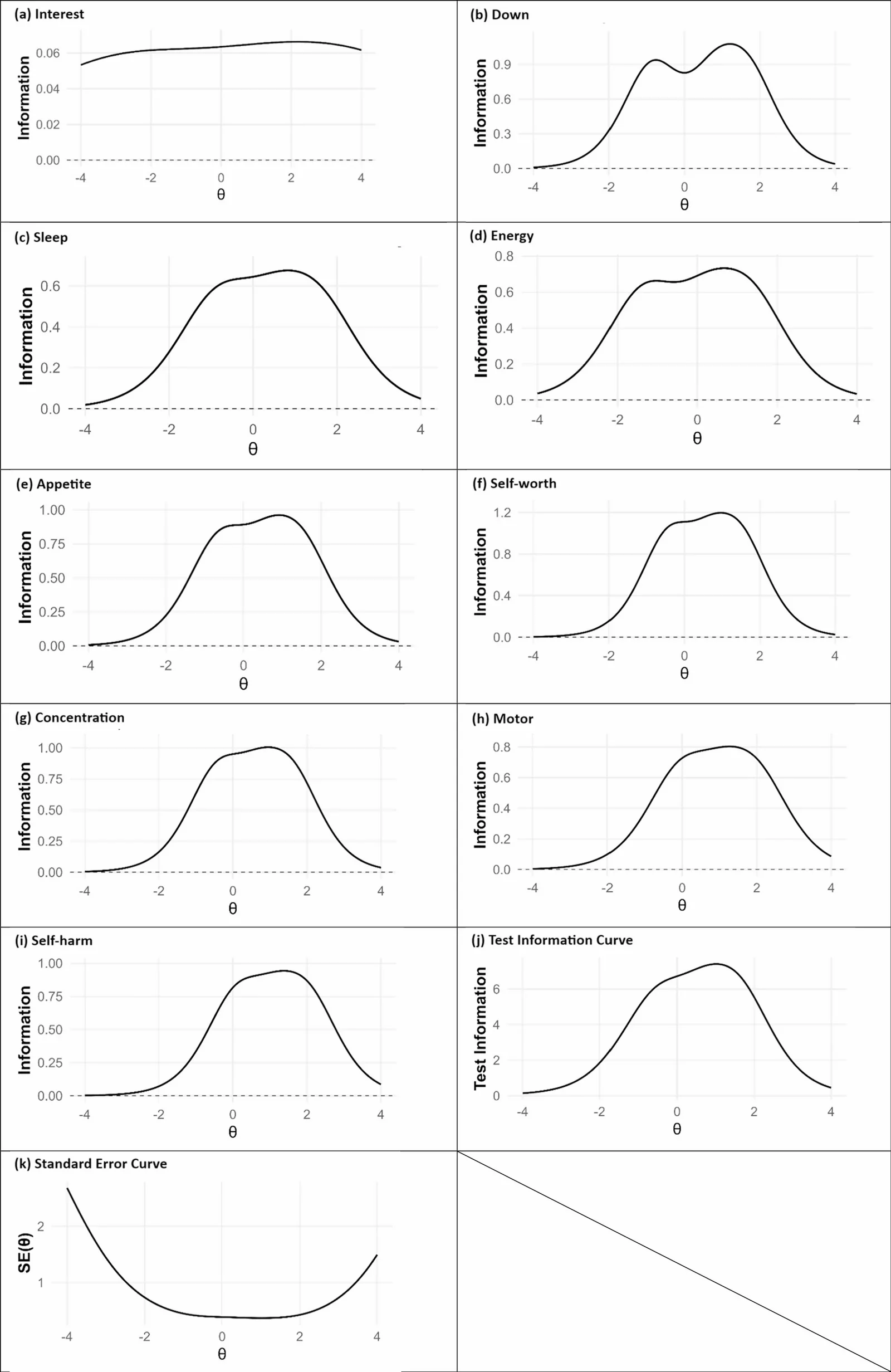
Item and test information functions for the PHQ-9 under the Graded Response Model. (a–i) Item information curves for PHQ-9 items 1–9, showing measurement information across levels of latent depression (θ). Panels correspond to (**a**) interest, **b** down, **c** sleep, **d** energy, **e** appetite, **f** self-worth, **g** concentration, **h** motor, and (**i**) self-harm. Items assessing self-worth, concentration, down, and appetite provide the highest information, whereas interest provides the least. **j** Test information curve for the PHQ-9, showing greatest overall measurement information between approximately θ = 0 and +2. **k** Standard error curve for the PHQ-9, showing the lowest standard error over approximately the same θ range, indicating greatest measurement precision in this region of the latent trait.

#### IRT item fit

Item-level fit statistics under the GRM revealed significant misfit for two items: self-worth (S-X² = 67.74, *p* = 0.005) and self-harm (S-X² = 65.12, *p* = 0.026). The remaining seven items showed adequate fit (all S-X² *p* > 0.12). The misfit of the self-worth and self-harm items likely reflects their severe symptom content and skewed response distributions in this non-clinical student sample; these items were retained for consistency with standard PHQ-9 administration and to preserve comparability with prior studies. Local dependence analysis identified elevated residual associations between self-harm and down (χ² = 43.16), self-harm and appetite (χ² = 45.02), and between several other item pairs (all χ² < 38), suggesting minor local dependence that does not substantially compromise overall model utility.

### Symptom network analysis

A partial correlation network of 30 items (9 PHQ-9 + 21 DASS-21) was estimated using graphical LASSO with the EBIC tuning procedure. Prior to network estimation, a Goldbricker redundancy check was performed to identify potentially overlapping items. No item pairs exceeded the correlation threshold (*r* > 0.50) for redundancy, supporting retention of all 30 nodes.

The estimated network revealed distinct clustering within depression, anxiety, and stress domains. Strength centrality was highest for D6_SelfWorth, D4_Down, D3_NoLookForward, and P7_Concentration. Betweenness centrality was highest for P6_SelfWorth, D7_Meaningless, and P3_Sleep, indicating these items bridge symptom clusters. Closeness centrality was highest for A7_Scared and D1_NoPositive, suggesting these symptoms have the shortest path to all other nodes in the network.

Bootstrapped correlation stability coefficients were CS-strength = 0.44, CS-betweenness = 0.31, and CS-closeness = 0.38, exceeding the recommended threshold of 0.25 for strength centrality. The network structure and centrality plots are shown in Fig. 4.

**Fig. 4:**
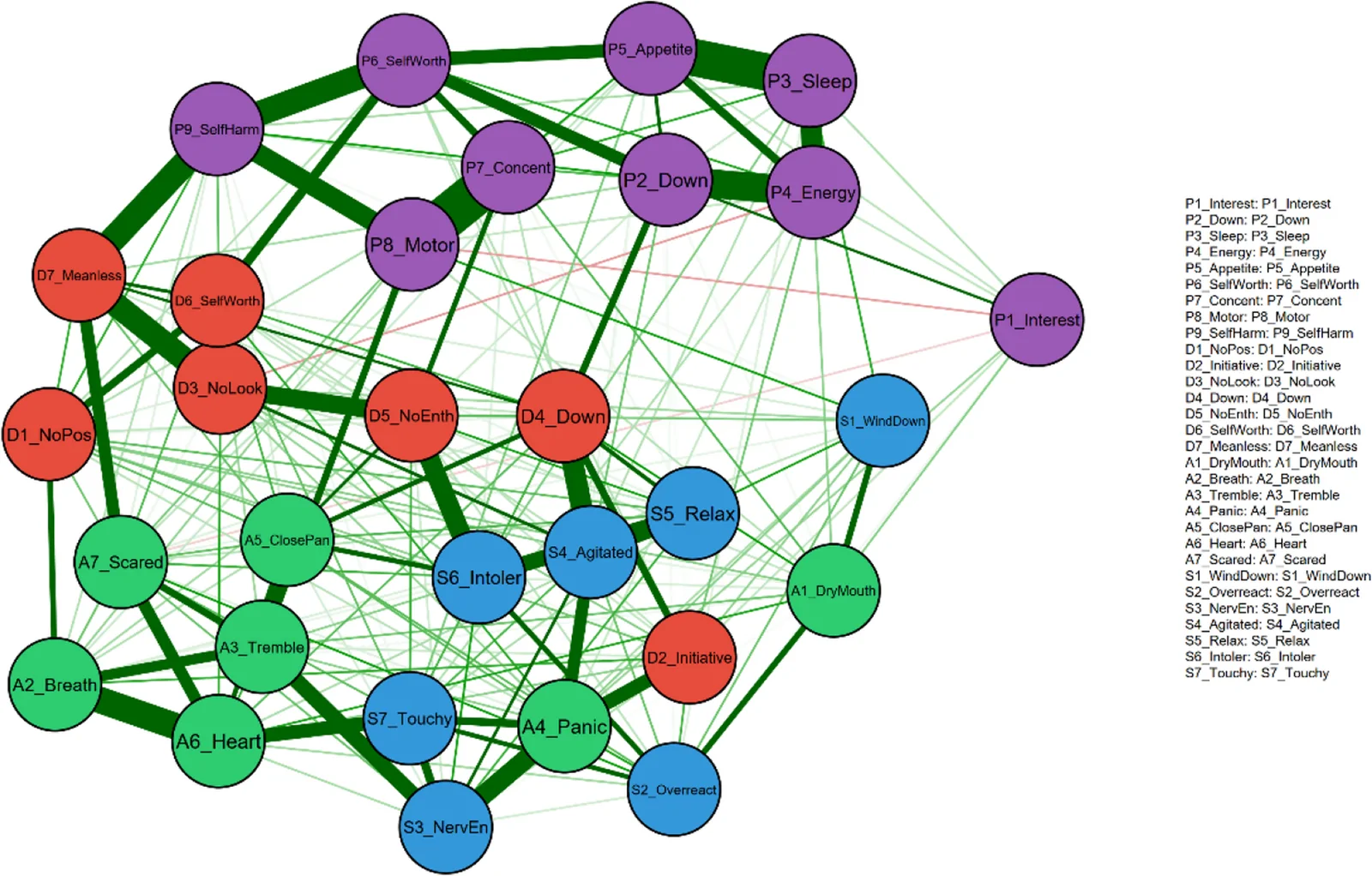
Symptom network analysis of PHQ-9 and DASS-21 items. Symptom network analysis of PHQ-9 and DASS-21 items (30 nodes). Network graph depicting partial correlations among items. Node colors represent domains (Depression = red, Anxiety = green, Stress = blue, PHQ-9 = purple). Edge thickness corresponds to association strength; green edges = positive, red edges = negative. Central nodes (self-worth, concentration, downheartedness) cluster strongly; bridging items (sleep, appetite, energy) connect domains

## Discussion

This study examined depressive and anxiety symptom differences between medical and non-medical university students in Pakistan, alongside a detailed psychometric evaluation of the PHQ-9 and DASS-21. Three findings warrant emphasis. First, non-medical students reported higher depressive and anxiety symptoms than medical students after adjustment a pattern that contradicts the assumption that medical training uniquely elevates distress. Second, both instruments performed adequately, though with important nuances: DASS-21 latent factor correlations approached unity, IRT discrimination varied substantially across items, and network centrality identified cognitive and self-worth symptoms as structurally central. Third, female gender and shorter sleep duration replicated as independent correlates, while treated depression history emerged as the single strongest predictor. These results carry implications for campus mental health policy and screening practice.

### Group differences: medical versus non-medical students

The literature comparing depressive symptoms across academic disciplines is genuinely mixed. Some studies report higher distress among medical students, attributing the elevation to curricular intensity and a training culture that socialises students into suppressing vulnerability. Others find no difference, and a smaller body of work including the present sample documents higher scores among non-medical students. Our finding that non-medical students scored higher on PHQ-9 and DASS-21 Depression and Anxiety subscales after covariate adjustment adds to this latter camp.

Several considerations temper this conclusion. The sample was convenience-based, collected at a single time point, without data on academic year, examination proximity, or socioeconomic background. Discipline may be confounded with unmeasured variables help-seeking norms, peer support access, or the stigma attached to reporting distress within medical training that influence both symptom expression and disclosure.

More fundamentally, discipline explains trivial variance. Adjusted R² values ranged from 0.029 to 0.041. Even the statistically significant differences represent a minuscule proportion of total variance in depression scores. In the fully adjusted model, treated depression history (β = 4.15, *p* < 0.001) vastly outweighed discipline effects. A prior clinical diagnosis carries roughly three times the predictive weight of academic field. Screening and intervention efforts should prioritise clinical history and current symptom burden over discipline-based triage.

The absence of a significant discipline difference on DASS-21 Stress (*p* = 0.089) is informative: the significant effects were confined to mood and anxiety domains, hinting that discipline differences may operate through dysphoric affect and worry pathways rather than perceived stress per se though the near-unity latent correlations among DASS-21 factors complicate any clean interpretation of subscale specificity.

### Gender and sleep as correlates of depression

Female students scored higher across all domains. This replicates a robust cross-national pattern: meta-analytic evidence from representative samples confirms a consistent female-male gap in depressive symptoms beginning in adolescence and persisting through midlife [10]. The magnitude here (β ≈ 1.7–2.2) is somewhat smaller than estimates from some Western samples, possibly reflecting cultural variation in symptom reporting or gendered help-seeking patterns in Pakistan. The consistency of the effect across depression, anxiety, and stress not its magnitude is what matters for practice: it supports targeted prevention and early outreach toward female students regardless of discipline.

Each additional hour of sleep was associated with a 0.37-point reduction in PHQ-9 score. A three-hour sleep difference maps onto roughly one severity category shift clinically meaningful at the population level. Cohort meta-analyses linking short sleep to incident depression support the plausibility of a directional effect [18, 19]. Reverse causation is equally plausible: depression fragments sleep architecture and prolongs sleep onset latency. The relationship is almost certainly bidirectional, and cross-sectional data cannot adjudicate directionality. Sleep duration nonetheless represents a modifiable, low-risk target hygiene programmes, scheduling adjustments during examination periods, and targeted outreach carry minimal downside and align with a growing evidence base for sleep-focused prevention [18, 19].

### Psychometric properties of the PHQ-9 and DASS-21

The PHQ-9 demonstrated acceptable internal consistency (α = 0.823), though this falls at the lower end of typical validation reports, where α commonly ranges from 0.86 to 0.89 [13, 20]. Whether this reflects genuine cultural variation in item interpretation for instance, differential attribution of somatic symptoms to physical illness versus depression or stems from sampling characteristics cannot be determined here. The instrument nonetheless supported valid group comparisons, and its brevity and DSM-concordant structure remain pragmatic advantages.

IRT analysis revealed marked heterogeneity in item discrimination. Self-worth, concentration difficulties, low mood, and appetite disturbance items showed the highest discrimination (a = 1.783–1.991), indicating these symptoms carry the most measurement information across the latent depression continuum. This aligns with prior graded response model studies showing cognitive and self-critical symptoms as the most informative indicators of depression severity [13, 21]. By contrast, the interest item (anhedonia) discriminated poorly (a = 0.468). Anhedonia may function differently in South Asian student populations, where reduced interest in activities can be attributed to contextual pressures examination schedules, family obligations, economic constraints rather than to depression per se [20]. Screening protocols that weight anhedonia heavily may underdetect cases in this population. Two items self-worth and self-harm showed statistically significant misfit under the GRM (both S-X² *p* < 0.05), likely reflecting their severe symptom content and floor effects in a non-clinical sample. This pattern is consistent with prior IRT studies of the PHQ-9 in analogue populations, where severe items often show poorer fit due to sparse endorsement at the highest response categories. The misfit does not invalidate the scale but suggests that brief screeners relying heavily on these items may perform less reliably at the severe end of the continuum in student samples.

The DASS-21 three-factor model achieved acceptable fit (CFI = 0.954; TLI = 0.948; RMSEA = 0.052), consistent with prior validations across diverse cultures [20, 22, 23]. However, the latent correlations demand scrutiny. Depression-Stress *r* = 0.939 and Anxiety-Stress *r* = 0.949 values approaching the reliability ceilings of the subscales. At this magnitude, the factors are not empirically distinguishable; a subscale cannot claim to measure a distinct construct when it shares over 88% of true-score variance with another. This pattern is consistent with validation studies in diverse cultural settings that have reported acceptable fit for bifactor and higher-order alternatives alongside the standard correlated-factors model [20, 22, 23], and it raises practical questions about whether the three DASS-21 subscales should be interpreted separately in Pakistani student samples. Researchers in this context should consider reporting a total score alongside subscales, or fitting a bifactor model to partition general distress from domain-specific variance. Against this backdrop, measurement invariance across academic discipline represents a genuine contribution: it supports valid comparison of medical and non-medical students on the DASS-21, even if the meaning of the subscale scores warrants cautious interpretation.

### Symptom network structure

Network analysis complemented the factor-analytic and IRT findings by identifying which symptoms occupy structurally central positions. Self-worth, concentration, and downheartedness showed the highest strength centrality, while self-worth, meaninglessness, and sleep problems exhibited the greatest betweenness serving as bridges across symptom domains. This supports the “centrality of cognitive symptoms” hypothesis that has emerged across multiple network studies of depression [24, 25]. Somatic items sleep, appetite, energy connected the mood/cognitive cluster with anxiety and stress, consistent with theoretical models positioning somatic symptoms as the interface between psychological distress and physiological dysregulation. Interest remained peripheral, suggesting anhedonia may operate as a downstream consequence rather than a core driver.

Stability coefficients (0.31–0.44) fell below the 0.50 ideal but exceeded the 0.25 minimum, indicating moderate robustness. These networks are exploratory and descriptive derived from cross-sectional data, non-regularised, and causally uninformative. Confirmatory approaches with pre-registered pipelines and temporal designs are needed to evaluate whether centrality structures replicate and whether central symptoms prospectively predict peripheral symptom emergence.

### Implications

Several findings carry actionable weight. The comparable or higher symptom burden among non-medical students challenges the assumption that medical training is uniquely toxic to mental health, supporting a whole-university approach contingent on replication in probability samples. The consistent female elevation supports gender-targeted prevention delivered early in the academic year. Sleep interventions remain low-risk and scalable, though the evidence base is stronger in prospective than cross-sectional designs. From a screening standpoint, the IRT and network findings converge practically: brief screeners prioritising cognitive and self-worth items will capture more measurement information, while anhedonia-focused items may underperform and require supplementation.

### Strengths and limitations

Strengths include the relatively large sample (*n* = 602), concurrent use of two validated instruments, and the application of multiple psychometric methods CFA, IRT, network analysis, and measurement invariance testing in combination, which exceeds the typical psychometric examination in student mental health research. Invariance testing across academic discipline is a particular strength, as it underpins the primary group comparison.

Limitations are substantial. The cross-sectional design precludes causal inference. Convenience sampling introduces selection bias; participants may differ systematically from non-respondents. Self-report instruments inflate shared-method variance. We lacked data on examination timing, socioeconomic status, and academic year. English-only administration may have excluded students with weaker English proficiency. The network analysis was exploratory and not regularised. Measurement invariance across sex was not tested, limiting confidence in the gender comparison. Two PHQ-9 items (self-worth and self-harm) exhibited statistically significant misfit under the graded response model, likely reflecting their severe symptom content and skewed endorsement in a non-clinical sample; future work should examine bifactor or two-tier IRT models to accommodate potential multidimensionality. The very high latent correlations and low R² values are not methodological flaws per se, but they constrain conclusions about DASS-21 subscale specificity and the practical significance of group differences.

## Conclusion

Non-medical students in this Pakistani sample reported higher depressive and anxiety symptoms than their medical counterparts after adjustment challenging the default assumption that medical training carries a uniquely heavy psychological burden and pointing toward the need for campus-wide mental health services. Both instruments performed adequately, though the DASS-21’s near-unity latent correlations call into question whether its three subscales measure distinguishable constructs here. Item-level analyses identified cognitive and self-worth symptoms as the most informative indicators of depression severity, while anhedonia contributed minimally a pattern with direct implications for screening item selection. What remains critically needed is longitudinal, representative data that can track symptom trajectories across academic years, test causal pathways, and determine whether the discipline differences and psychometric properties observed here generalise beyond convenience samples.

## Supplementary Information

Supplementary Material 1.

Supplementary Material 2.

Supplementary Material 3.

Supplementary Material 4.

Supplementary Material 5.

Supplementary Material 6.

Supplementary Material 7.

Supplementary Material 8.

Supplementary Material 9.

Supplementary Material 10.

Supplementary Material 11.

Supplementary Material 12.

Supplementary Material 13.

## Data Availability

The datasets generated and/or analysed during the current study are available from the corresponding author on reasonable request.
