# Supplementary material for: Psychometric properties and symptom profiles of the PHQ-9 and DASS-21 among medical and non-medical university students: a cross-sectional study in Pakistan
Source: BMC Psychol. 2026 Aug 12;14:1396. doi: 10.1186/s40359-026-05332-5 (PMC13617949; doi:10.1186/s40359-026-05332-5)
Supplement: Supplementary file 1 [file 40359_2026_5332_MOESM1_ESM.docx]

**1. Group Comparisons (Wilcoxon rank-sum, Chi-square tests)**

- Needed to check whether baseline demographic and lifestyle variables differed between medical and non-medical students.
- Nonparametric tests used to avoid assumptions of normality.

**2. Symptom Severity (PHQ-9, DASS-21 categories)**

- Frequency distributions allow intuitive understanding of prevalence/severity patterns.
- Clinical categories help contextualize findings beyond raw scores.

**3. Regression Analysis**

- Multiple regression identifies predictors of depression scores while controlling for confounders (gender, age, sleep).
- Robust standard errors used to ensure reliable inference.

**4. Confirmatory Factor Analysis (CFA) & Invariance Testing**

- Essential for validating whether DASS-21’s 3-factor structure fits in this sample.
- Invariance ensures fair comparisons across medical vs. non-medical groups.

**5. Reliability (Cronbach’s α)**

- Demonstrates internal consistency, ensuring scales are psychometrically sound.

**6. Item Response Theory (IRT)**

- Provides deeper item-level insights into PHQ-9, identifying which items best detect severity differences.
- Shows test precision across the depression continuum (mild → severe).

**7. Symptom Network Analysis**

- Explores interconnections between depression, anxiety, and stress symptoms.
- Helps identify “central” or “bridge” symptoms that might explain comorbidity.
